# Myelination and Remyelination in Peripheral Nerve Injury: Mechanisms, Regulation, and Therapeutic Perspectives

**DOI:** 10.1002/mco2.71019

**Published:** 2026-10-11

**Authors:** Li‐Xin Huang, Tao Sun, Yi‐Bo Zhao, Zhi‐Min Wu, Jun Sun, Ming‐Yang Li, Qing‐Yi Huo, Yun‐Mou Ou, Qing‐Tian Liang, Cong Ling, Bao‐Yu Zhang, Chuan Chen, Ying Guo, Hui Wang

**Affiliations:** ^1^ Department of Neurosurgery The Third Affiliated Hospital Sun Yat‐Sen University Guangzhou China

**Keywords:** myelination, peripheral nerve injury, remyelination, Schwann cell, signal transduction

## Abstract

Peripheral nerve injury (PNI) affects 13–23 individuals per 100,000 annually, and functional recovery hinges on faithful remyelination of regenerating axons by Schwann cells (SCs). This review integrates the complete molecular logic governing the transition from injury‐induced SC plasticity to mature myelin regeneration. The hierarchical transcriptional program—from SOX10 pioneer factor engagement through OCT6‐mediated promyelinating transition to KROX20/EGR2‐driven activation of compact myelin genes—and the instructive role of axonal Neuregulin‐1 Type III signaling are dissected. Wallerian degeneration via the NMNAT2–SARM1‐mediated axon degeneration cascade, c‐JUN‐dependent SC reprogramming, and the epigenetic and noncoding RNA mechanisms that re‐engage myelination are examined. The immune microenvironment is considered as a temporally layered network in which macrophage polarization, macrophage‐to‐myofibroblast transition, and regulatory T cell activity coordinate the transition from inflammation to repair. Finally, this review critically evaluates emerging therapeutic strategies—including Neuregulin‐1 supplementation, SC transplantation, nerve guidance conduits, electrical stimulation, and senolytic clearance of aged SCs—and argues that rationally sequenced combination therapies will be required to achieve transformative improvements in functional recovery after severe PNI. This review provides a mechanistic roadmap for designing next‐generation regenerative strategies aimed at restoring saltatory conduction.

## Graphical abstract

Peripheral nerve injury triggers axonal degeneration, myelin breakdown, and Schwann cell reprogramming. Remyelination requires reactivation of the SOX10–OCT6–KROX20/EGR2 transcriptional hierarchy, axonal NRG1–ErbB2/3 signaling, metabolic support, and coordinated immune regulation. Emerging biological, biomaterial, biophysical, and pharmacological strategies target distinct stages of this cascade, with rationally sequenced combination therapies offering a promising route toward functional recovery.

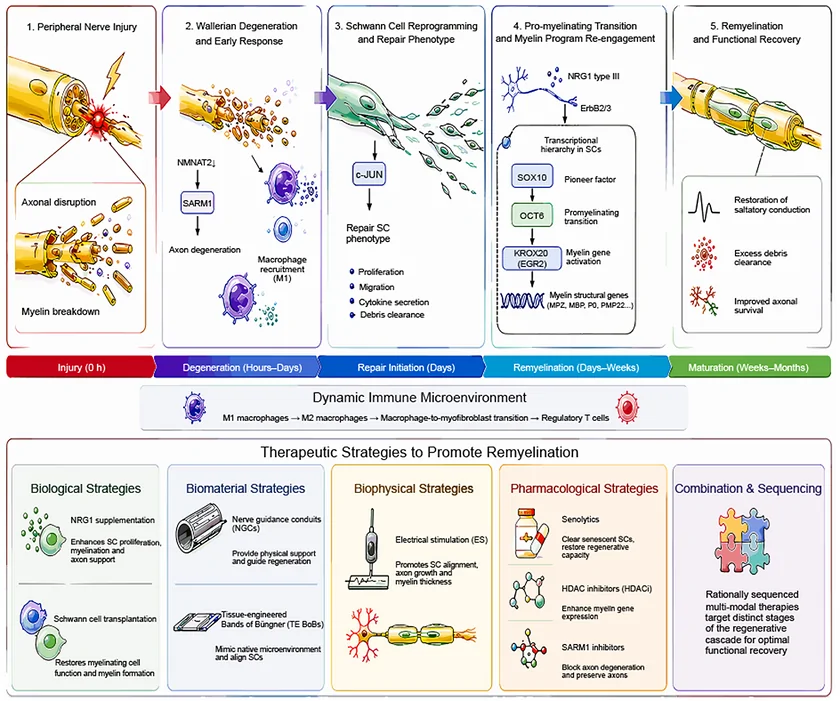

## 1 Introduction

Peripheral nerve injuries (PNIs) affect approximately 13–23 per 100,000 individuals per year, imposing a major socioeconomic burden through long‐term disability and the high costs of surgical and rehabilitative care [1, 2]. Unlike axons in the central nervous system (CNS), peripheral axons retain an intrinsic capacity for regeneration; yet clinical outcomes frequently remain unsatisfactory, particularly after severe or delayed injuries when the distal nerve stump undergoes progressive denervation atrophy before regenerating axons arrive [3].

Central to successful functional recovery is the re‐establishment of myelin sheaths around regenerating axons. Myelin, produced in the peripheral nervous system (PNS) by Schwann cells (SCs), is a specialized multilamellar lipid membrane that enables rapid, metabolically efficient saltatory conduction [4]. Loss of myelin following injury causes conduction slowing, conduction block, and—if remyelination fails—ultimately irreversible axonal degeneration. The transcriptional cascade governing SC myelination has been substantially elucidated through knockout and lineage‐tracing mouse studies: SOX10‐expressing SC precursors progress through OCT6‐positive promyelinating cells to KROX20/EGR2‐driven myelinating SCs [5, 6, 7, 8, 9, 10, 11, 12]. After injury, this cascade reverses and must later re‐engage, a process orchestrated by the transcription factor c‐JUN and the so‐called repair SC phenotype [13, 14, 15].

Despite these molecular advances, functional outcomes after severe PNI remain suboptimal, largely because remyelination in humans is markedly slower than in rodents, surgical delays compound distal stump atrophy, and aging imposes additional epigenetic and cellular barriers. This review examines the structural and functional properties of peripheral myelin, the transcriptional and epigenetic control of SC identity, the cellular events of Wallerian degeneration and SC reprogramming, emerging immune mechanisms of nerve repair, and current and emerging therapeutic strategies aimed at accelerating remyelination to improve functional outcomes after PNI.

The molecular determinants of remyelination success are organized around four interdependent axes: (i) the transcriptional and epigenetic program that establishes and re‐engages SC identity, from the SOX10–OCT6–KROX20/EGR2 activating backbone to the c‐JUN‐driven repair phenotype; (ii) the lipid and cholesterol metabolic machinery supplying the biosynthetic building blocks for new myelin; (iii) the innate immune microenvironment governing clearance of inhibitory myelin debris; and (iv) the adaptive immune system, principally T lymphocytes, that fine‐tunes the transition from inflammation to repair. Together with the intervening cellular events of Wallerian degeneration and the barriers imposed by aging and metabolic disease, these axes provide the mechanistic scaffold on which the combination therapies discussed in later sections are built.

## 2 Peripheral Myelin: Structure, Composition, and Function

Peripheral myelin is generated by the spiral wrapping of the SC plasma membrane around the axon, producing the stack of compact membrane layers visible in cross‐section (Figure 1A) [16]. Along the internode, this compact sheath is interrupted at regular intervals by the node of Ranvier, flanked by paranodal regions where glial loops attach to the axolemma, and by noncompact, cytoplasm‐rich Schmidt–Lanterman incisures that provide a radial conduit for metabolite and ion exchange across the sheath (Figure 1B). This function is mediated in part by Connexin 32 (Cx32)‐containing reflexive gap junctions, whose loss causes X‐linked Charcot–Marie–Tooth disease (CMTX) [17]. The ordered assembly, disassembly, and reassembly of this architecture are hallmarks of Wallerian degeneration and remyelination after PNI, making its molecular basis directly relevant to the mechanisms addressed throughout this review [18, 19, 20].

**FIGURE 1:**
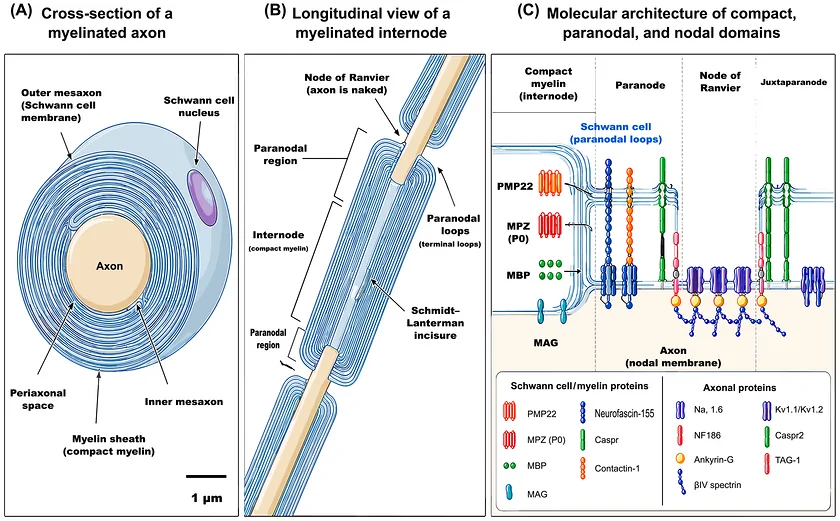
Structural organization of the peripheral myelinated nerve fiber. (A) Cross‐sectional view of a myelinated peripheral axon illustrating concentric compact myelin lamellae formed by Schwann cell membrane wrapping. The outer and inner mesaxons, periaxonal space, major dense line, and intraperiod line are indicated. (B) Longitudinal view of a myelinated internode showing the node of Ranvier, paranodal terminal loops, juxtaparanodal region, compact internodal myelin, and Schmidt–Lanterman incisures. The nodal axolemma is exposed and flanked by paranodal loops formed by Schwann cells. (C) Molecular architecture of compact and noncompact myelin domains illustrating the spatial distribution of major structural and functional proteins in compact myelin, paranodes, nodes of Ranvier, and juxtaparanodes. Abbreviations: AnkG, ankyrin‐G; Caspr, contactin‐associated protein‐1; Caspr2, contactin‐associated protein‐2; Cntn1, contactin‐1; Cx32, Connexin 32; MAG, myelin‐associated glycoprotein; MBP, myelin basic protein; MPZ (P0), myelin protein zero; Nav1.6, voltage‐gated sodium channel 1.6; NF155, Neurofascin‐155; NF186, Neurofascin‐186; PMP22, peripheral myelin protein 22; TAG‐1, transient axonal glycoprotein‐1.

The molecular organization of compact and noncompact myelin is summarized in Figure 1C. Compact myelin protein is dominated by myelin protein zero (MPZ/P0), comprising 50–70% of total PNS myelin protein; P0 is a transmembrane immunoglobulin‐superfamily protein that acts as a tetrameric homophilic adhesion molecule, with its basic cytoplasmic domain stabilizing intracellular leaflet adhesion, while myelin basic protein (MBP) neutralizes the negatively charged cytoplasmic membrane surface to complete compaction of the major dense line [16, 21]. Mutations in MPZ cause CMT1B and the more severe Dejerine–Sottas syndrome. Peripheral myelin protein 22 (PMP22) constitutes only 2–5% of myelin protein but is strictly dosage‐sensitive: trisomy causes CMT1A, heterozygous deletion causes hereditary neuropathy with liability to pressure palsies, and point mutations cause CMT1E; PMP22 dosage imbalance also independently disrupts SC cholesterol and lipid‐raft homeostasis [22]. Lipids—chiefly galactosylceramide, sulfatide, cholesterol, and plasmalogens—constitute roughly 70% of myelin dry weight [23]. Cholesterol is not merely structural: SC‐specific loss of cholesterol biosynthesis causes severe hypomyelination, because P0 export from the endoplasmic reticulum to the myelin membrane is itself cholesterol‐dependent, directly coupling lipid availability to the pace of myelin assembly [24, 25, 26, 27, 28, 29, 30].

At the node of Ranvier, high densities of voltage‐gated sodium channels (primarily Nav1.6), Na^+^/K^+^‐ATPase, and Na^+^/Ca^2^^+^ exchangers generate the regenerative currents that drive saltatory conduction; Nav1.6 is anchored to the nodal membrane through ankyrin‐G and βIV spectrin, which link the channel complex to the submembranous actin cytoskeleton [31]. Paranodal junctions flanking each node are formed by a tripartite complex of axonal contactin and contactin‐associated protein (Caspr/paranodin) interacting with glial Neurofascin‐155 (NF155), which acts both as an electrical seal and a structural anchor separating nodal Na^+^ channels from juxtaparanodal K^+^ channels [32]. Recent ultrastructural and electrophysiological evidence further indicates that the periaxonal space beneath the myelin sheath behaves as a leaky, capacitively coupled nanocircuit rather than a fully sealed cable, adding a further layer of complexity to how internodal geometry shapes conduction [18]. Following PNI, paranodal detachment and nodal lengthening are among the earliest structural correlates of conduction failure, and the fidelity with which Nav1.6 clustering and paranodal junctions are re‐established during SC‐mediated remyelination is now recognized as both a structural prerequisite for, and a measurable readout of, functional nerve recovery (Figure 1).

## 3 Transcriptional Regulation of SC Development and Myelination

### 3.1 Overview of the Transcriptional and Epigenetic Landscape

SC myelination is not governed by a single master switch but by a temporally ordered, hierarchical network of transcriptional activators, corepressors, and chromatin‐remodeling complexes that must be induced, silenced, and re‐induced in strict sequence. Three classical factors, SOX10, OCT6/POU3F1, and KROX20/EGR2, form the activating backbone of this network, converting axonal signals into coordinated expression of the myelin structural gene program [5, 9, 11] (Table 1). This activating axis, however, operates within a substantially broader regulatory landscape that has expanded considerably in recent years. Corepressor and chromatin‐remodeling complexes—including NAB1/2‐nucleosome remodeling and deacetylase (NuRD), the zinc‐finger repressor Zeb2, and the Polycomb Repressive Complex 2 (PRC2)—actively silence competing lineage programs and gate chromatin accessibility at myelin and injury‐response loci. Mechanotransductive and metabolic effectors, notably the Hippo‐pathway coactivators YAP/TAZ and the transcription factor MAF, couple physical and lipidomic cues to this core program. Superimposed on the developmental network is an injury‐inducible transcriptional switch centered on c‐JUN, which reprograms myelinating SCs into a distinct “repair” phenotype after PNI.

**TABLE 1:** Key transcriptional regulators of SC myelination and remyelination.

Factor	Expression phase	Role in myelination	Effect of loss‐of‐function	References
SOX10	Precursor → mature SC	Pioneer factor; activates myelin genes; maintains SC lineage	SC lineage failure; severe peripheral neuropathy	[73]
OCT6/SCIP	Promyelinating SC	Activates KROX20 expression; promotes transition to myelinating SC	Transient myelination delay (compensated by KROX20)	[74]
KROX20/EGR2	Myelinating SC	Master activator of compact myelin genes; represses immature markers	Complete failure of myelination (severe neuropathy)	[48]
c‐JUN	Repair SC (postinjury)	Promotes repair SC phenotype; activates trophic factors; inhibits KROX20	Impaired axon regeneration; failed remyelination	[75]
YY1	Myelinating SC	Activates myelin gene transcription; cooperates with SOX10	Impaired myelination; neuropathy	[76]
HDAC1/2	Myelinating SC (remyelination)	Epigenetic silencing of immature SC genes	Failure of KROX20 upregulation; hypomyelination	[77]

Abbreviations: c‐JUN, cellular Jun proto‐oncogene (AP‐1 transcription factor subunit); EGR2, early growth response 2; HDAC1/2, histone deacetylase 1/2; KROX20, Krüppel‐like zinc‐finger protein 20 (synonym for EGR2); OCT6, octamer‐binding transcription factor 6; SC, Schwann cell; SCIP, suppressed cAMP‐inducible POU protein (synonym for OCT6); SOX10, SRY‐related HMG‐box 10; YY1, Yin Yang 1.

### 3.2 SOX10: Pioneer Factor and Lineage Maintenance

SC differentiation into mature myelinating cells is orchestrated by a hierarchical transcriptional network. At its apex is SOX10, a SRY‐related HMG‐box transcription factor highly expressed in emerging neural crest and developing PNS [33]. Loss of Sox10 in mice causes complete absence of peripheral glia, and conditional deletion in immature SCs causes death within the seventh postnatal week from peripheral neuropathy; in adult mice, Sox10 loss in mature SCs triggers demyelination and axonal degeneration, demonstrating that SOX10 is required not only for differentiation but for continuous maintenance of the differentiated state [34]. At the chromatin level, SOX10 functions as a genuine pioneer factor: genome‐wide ChIP‐seq analysis of myelinating peripheral nerve demonstrates that SOX10 independently activates a large fraction of myelination genes including Oct6, Egr2/Krox20, Mpz, Mbp, Pmp22, and ErbB3 [35]. Following nerve injury, SOX10 protein levels decrease but do not disappear in repair SCs, potentially ensuring that the capacity for future remyelination is retained within the dedifferentiated SC pool [36, 37].

### 3.3 OCT6 And the Myelination Checkpoint

The transition from immature SC to the promyelinating state is controlled by the POU‐domain transcription factor OCT6 (also known as SCIP or Tst‐1), which functions as a regulatory keystone linking upstream SOX10 activity to downstream induction of KROX20/EGR2 [38, 39]. Ghislain and Charnay identified the myelinating SC element within the Mpz gene, a cis‐regulatory element integrating OCT6, BRN2, and SOX10 activities in a composite structure, suggesting that cooperative factor binding on shared regulatory elements is the molecular basis for high‐level myelination gene expression [40, 41]. Mice with homozygous deletion of Oct6 exhibit a transient but ultimately compensated arrest at the promyelinating stage, because the closely related POU factor BRN2 (POU3F2) can belatedly substitute for OCT6 [42]. This partial redundancy explains why Oct6 loss produces a milder phenotype than Sox10 or Krox20 loss and suggests that multiple POU factors maintain the OCT6→EGR2 regulatory axis during regeneration [43].

### 3.4 KROX20/EGR2: Master Activator of the Myelin Gene Program

Among all myelination transcription factors, KROX20/EGR2 occupies a singular, nonredundant position. Its expression in SCs is strictly confined to the myelinating stage, and loss of KROX20 results in complete arrest of myelination, demonstrating that it is required not only for initiation but also for maintenance of the myelinating state in adult nerves [44]. KROX20 activity requires coregulatory factors including NAB1 and NAB2 (NGFI‐A/Egr binding) core repressors, which recruit the NuRD complex [45, 46, 47]. The NuRD catalytic subunit CHD4 interacts with NAB core repressors and is required for peripheral nerve myelination; Chd4 conditional deletion in SCs produces delayed myelination, radial sorting defects, hypomyelination, and persistent promyelinating SCs without affecting KROX20 levels themselves, indicating CHD4 acts downstream of EGR2 induction [48, 49, 50]. An antisense RNA transcribed from the EGR2 promoter (EGR2‐AS) has emerged as a regulatory layer that recruits activating and repressive histone marks to c‐JUN and EGR2 promoters respectively, orchestrating epigenetic switching between myelinating and repair SC states [51]. Clinically, dominant and recessive EGR2 mutations cause phenotypes ranging from congenital hypomyelinating neuropathy through CMT1D to Dejerine–Sottas syndrome [52, 53].

### 3.5 Synergistic Action of SOX10 and KROX20

The partnership between SOX10 and KROX20 is synergistic and mechanistically essential [36, 37, 54]. Genome‐wide ChIP‐seq analysis reveals substantial colocalization of EGR2 and SOX10 binding at multiple regulatory elements. While SOX10 itself regulates Egr2/Krox20 expression, leading to synergistic activation of major myelin genes, a substantial fraction of genes are activated by SOX10 independently of EGR2, and conversely, a set of SOX10‐dependent early SC genes is subsequently repressed by EGR2/KROX20 as cells progress to the myelinating state [35]. This genomic analysis reveals a temporal logic in which the chromatin‐opening pioneer activity of SOX10 precedes and enables KROX20 binding, after which the two factors interact on composite elements to achieve the transcriptional magnitude required for synthesizing vast quantities of myelin [39, 55].

Among the negative regulators that must be actively repressed during myelination, c‐JUN, SOX2, NOTCH, and ID2 are principal factors maintaining immature or repair SC identity and are directly repressed by KROX20 [56, 57, 58]. YY1 acts as a positive effector linking NRG1 signaling to the transcriptional myelination program: the neuregulin–ErbB2/3‐ERK1/2‐YY1 axis regulates Egr2 transcription, and injury causes a significant reduction in phospho‐YY1 leading to loss of Egr2 transcription [59, 60, 61].

### 3.6 Beyond the Classical Triad: Recently Identified Regulators

Genetic and genomic studies over the past decade have substantially extended the transcriptional network beyond SOX10, OCT6, and KROX20, revealing at least four additional layers of control that are directly relevant to PNI and remyelination. The zinc‐finger repressor Zeb2 acts as a critical intrinsic timer of SC differentiation by recruiting HDAC1/2–NuRD corepressor complexes to silence maturation inhibitors such as Sox2 and the Notch effector Hey2. Zeb2‐deficient SCs fail to sort axons or synthesize myelin, and Zeb2 deletion in adult mice both accelerates dedifferentiation into repair cells after PNI and impairs subsequent remyelination, identifying Zeb2 as a bidirectional switch operating at both the developmental and injury‐repair transitions [62, 63, 64]. Furthermore, the PRC2, which catalyzes repressive histone H3K27 trimethylation, restrains mature myelin thickness and keeps the SC injury‐response program—including Shh and Igfbp2—epigenetically silenced in the absence of injury. Loss of the PRC2 subunit Eed causes progressive hypermyelination and derepression of injury genes without activating c‐JUN itself, indicating that PRC2 sets a chromatin threshold that must be overcome for the repair program to launch [65, 66, 67]. And the Hippo‐pathway coactivators YAP and TAZ are activated in SCs by mechanical stimuli from the axon and basal lamina and are required for radial sorting and the transcription of basal‐lamina receptor genes during development [68]; after nerve crush injury, YAP/TAZ are additionally required for SC dedifferentiation, proliferation, and remyelination, extending their role from a purely developmental mechanosensor to an injury‐responsive regulator [69, 70, 71].

Subsequently, MAF links growth‐factor signaling to myelin lipid synthesis. The transcription factor Maf acts downstream of axonal Neuregulin‐1–ErbB signaling to drive maximal expression of cholesterol‐synthesis genes, a rate‐limiting step in myelin membrane growth; loss of Maf in myelinating SCs produces thin myelin despite normal induction of Egr2, showing that a dedicated transcriptional arm couples growth‐factor signaling to the lipogenic demands of myelination independently of the core Sox10–Egr2 axis [72]. In addition, c‐JUN plays an important role as the main switch for repairing SC. Following PNI, the transcription factor c‐JUN is rapidly induced in SCs and acts as a global regulator of Wallerian degeneration, governing the expression of trophic factors and adhesion molecules, the formation of Büngner‐band regeneration tracks, and myelin clearance. SC‐specific loss of c‐JUN produces a dysfunctional repair cell, failure of functional recovery, and neuronal death, formally establishing c‐JUN as the transcriptional counterpart of SOX10–EGR2 for the injury/repair state rather than a redundant or minor regulator [13]. Because c‐JUN both antagonizes EGR2‐driven myelin gene expression and is itself repressed by KROX20 during active myelination, the SOX10–EGR2 and c‐JUN programs constitute reciprocal, injury‐toggled states rather than independent pathways.

Having established how SOX10, OCT6, and KROX20/EGR2 build the mature myelinating SC, the following section examines how this machinery responds when the ensheathed axon is injured. The focus then turns to the acute reversal of this program—Wallerian degeneration and reprogramming into the repair SC phenotype.

## 4 Wallerian Degeneration and SC Reprogramming After Nerve Injury

### 4.1 Molecular Execution of Axon Degeneration: The NMNAT2–SARM1‐Mediated Axon Degeneration Cascade

Following axonal injury, the distal nerve stump undergoes stereotyped and molecularly programmatic degeneration first described by Augustus Waller in 1850 [78, 79, 80]. Modern genetics has delineated a canonical pathway in which the unstable axonal NAD synthase NMNAT2 functions as a survival factor whose loss is the proximal trigger for degeneration. After axotomy, NMNAT2 is rapidly degraded before axon fragmentation; genetic deletion of SARM1 completely rescues Nmnat2‐null embryonic lethality, indicating that NMNAT2 maintains axon integrity by suppressing the prodegenerative SARM1 protein [81]. The mechanistic cascade proceeds as follows: SARM1 possesses enzymatic NAD‐cleaving activity; following nerve injury and NMNAT2 depletion, accumulation of the NMNAT2 substrate NMN allosterically activates SARM1–NADase activity, driving NAD depletion and ultimately axon‐intrinsic calcium elevation—the final executioner event of axon fragmentation [82]. This pathway has attracted intense translational interest: SARM1 gain‐of‐function variants are enriched in sporadic amyotrophic lateral sclerosis and hereditary spastic paraplegia patients, and pharmacological or genetic SARM1 inhibition is robustly protective in multiple rodent peripheral neuropathy models [83]. This discovery has fundamentally repositioned Wallerian degeneration as an active, genetically encoded self‐destruction program—the NMNAT2–SARM1‐mediated axon degeneration cascade—analogous to apoptosis, with significant implications for therapeutic intervention within hours of PNI [84].

### 4.2 c‐JUN‐Driven Reprogramming: The Repair SC

The reprogramming of myelinating SCs following injury is orchestrated by the transcription factor c‐JUN, whose expression is rapidly upregulated after nerve injury through convergent activation of MAP kinase and JNK signaling pathways triggered by loss of axonal NRG1 signaling, mechanical SC stretching upon axon fragmentation, and cytokine receptor signaling from infiltrating immune cells [85]. c‐JUN is both necessary and sufficient for the repair SC phenotype: SC‐specific c‐JUN knockout mice exhibit severely impaired myelin clearance, disorganized Büngner band formation, reduced macrophage recruitment, and poor functional recovery after sciatic nerve crush [85, 86]. Conversely, SC‐specific c‐JUN overexpression accelerates the repair program, upregulating p75NTR, GDNF, BDNF, and artemin [87, 88, 89, 90].

A critical and frequently underappreciated feature of c‐JUN dynamics is its temporal decline during chronic denervation. RNA‐sequencing data show that c‐Jun and its target p75NTR peak at 1 week after injury and gradually decline during prolonged denervation; chronic 10‐week denervation causes significantly reduced motor neuron regeneration into denervated stumps, and this deficit is substantially rescued by restoring SC c‐JUN expression, even in aged mice [91, 92, 93]. This finding establishes the time‐dependent decline of c‐JUN as a key molecular mechanism underlying poor outcomes after delayed nerve repair and positions c‐JUN restoration as a tractable therapeutic target.

An important conceptual advance has been the discovery that mTORC1 plays an early, paradoxical role in the injury response before its later function in remyelination. mTORC1 is transiently but robustly reactivated in dedifferentiating SCs after nerve crush; inducible deletion of the mTORC1‐subunit Raptor in adult SCs impairs myelin clearance and SC dedifferentiation without major alterations in the inflammatory response, consequently delaying remyelination [94, 95, 96]. This establishes a biphasic role for mTORC1—first promoting dedifferentiation and debris clearance, then driving remyelination anabolism—as a central metabolic governor of the entire repair sequence.

### 4.3 Repair SC Function: Myelin Clearance, Immune Coordination, and Büngner Bands

Repair SCs perform multiple interdependent functions during Wallerian degeneration. They phagocytose myelin debris through macrophage‐like mechanisms including LC3‐associated phagocytosis and myelinophagy, a specialized form of SC‐intrinsic autophagy [97, 98, 99]. The biological necessity of this clearance is absolute: myelin debris contains potent inhibitors of axon regeneration (MAG, Nogo‐A, OMgp) and its persistence in chronically denervated or aging nerves is a principal cause of regeneration failure. SC‐derived chemokines (CCL2, CXCL1) and cytokines (LIF, IL‐6) recruit circulating monocytes that differentiate into endoneurial macrophages and amplify myelin phagocytosis through complement‐mediated and receptor‐mediated mechanisms [100, 101].

The most striking morphological consequence of repair SC reprogramming is the formation of Büngner bands. Using genetic lineage tracing and scanning block‐face electron microscopy, repair cells have been shown to be two–three times longer than myelinating and Remak cells and 7–10 times longer than immature SCs [97]. These cells provide physical guidance for regenerating axons, secrete neurotrophic factors (GDNF, BDNF, artemin) to sustain neuronal survival and stimulate axon elongation, and deposit extracellular matrix (ECM) proteins including laminin and Type IV collagen to stabilize the regeneration niche [102] (Figure 2).

**FIGURE 2:**
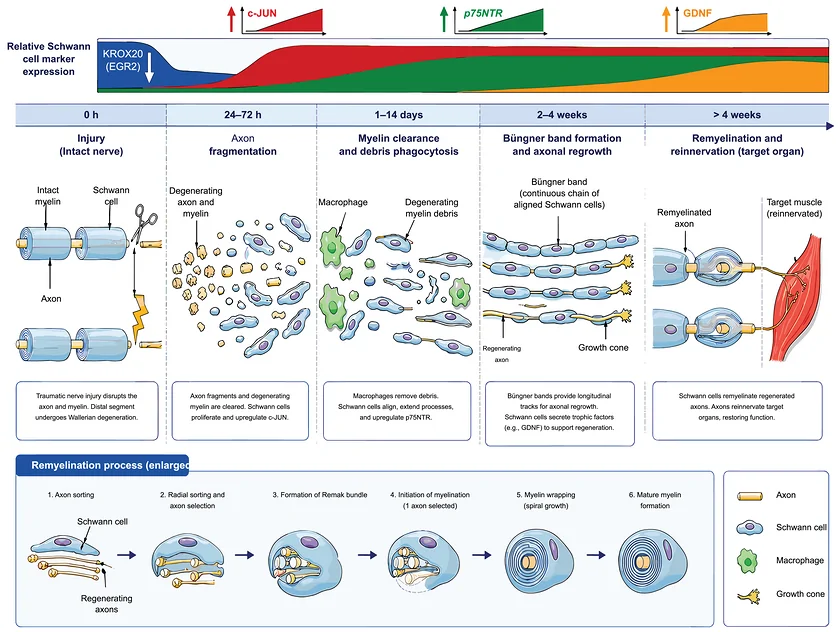
Temporal sequence of Wallerian degeneration, Schwann cell reprogramming, and remyelination following peripheral nerve injury. Schematic illustration of the sequential cellular and molecular events after peripheral nerve injury. Following axonal transection (0 h), distal axons undergo fragmentation within 24–72 h. During Days 1–14, degenerating myelin is removed by repair Schwann cells and infiltrating macrophages. Between approximately Days 7 and 28, repair Schwann cells align into continuous end‐to‐end Büngner bands, providing longitudinal guidance pathways for regenerating axons. Regenerating axons subsequently re‐enter distal nerve segments and initiate remyelination, followed by progressive maturation of myelin sheaths and functional target organ reinnervation during Weeks 4–12. The lower panel illustrates the sequential stages of remyelination, including Schwann cell alignment, axonal selection, initiation of myelin wrapping, spiral membrane growth, and mature compact myelin formation. Dynamic changes in Schwann cell‐associated gene expression are summarized above the timeline, including KROX20 (EGR2) downregulation immediately after injury, transient c‐JUN upregulation during repair, increased p75NTR and GDNF expression during regeneration, followed by c‐JUN downregulation, transient OCT6 re‐expression, and restoration of KROX20 during remyelination. Abbreviations: GDNF, glial cell line‐derived neurotrophic factor; KROX20 (EGR2), early growth response protein 2; OCT6, POU Class 3 homeobox 1; p75NTR, p75 neurotrophin receptor.

The repair SC is not an end state but a preparatory one: its debris clearance and Büngner band construction exist to permit a second, forward‐directed program. The next section returns to the molecular control of myelination, examining how the repair phenotype is suppressed and the developmental cascade re‐engaged.

## 5 Molecular Control of Remyelination in the PNS

### 5.1 Re‐engagement of the Myelination Cascade and the Role of NRG1

Once regenerating axons contact repair SCs, the remyelination program is initiated. This transition requires active suppression of the repair SC transcriptional program, principally through downregulation of c‐JUN, and sequential re‐engagement of the developmental myelination cascade: SOX10 activity is maintained throughout; OCT6 is transiently re‐expressed during the promyelinating phase; and KROX20/EGR2 is reactivated to drive compact myelin gene expression [36, 37]. The molecular trigger is again NRG1 Type III, whose re‐establishment on regenerating axon surfaces reactivates ErbB2/ErbB3→PI3K–AKT signaling in apposing SCs. Persistent ERK activation—as occurs in inflammatory neuropathy or CMT1A due to NRG1 Type I autocrine signaling in onion bulbs—impairs remyelination, and pharmacological MEK inhibition has been explored as a strategy to accelerate remyelination in demyelinating neuropathy models [103].

### 5.2 mTORC1 As a Quantitative Regulator of Myelin Production

A particularly important downstream mediator of PI3K–AKT in remyelination is mTORC1. mTORC1 promotes protein synthesis, lipid biosynthesis via SREBP transcription factors, and ribosome biogenesis required for the massive anabolic expansion of SCs during myelination. SC‐specific conditional deletion of Raptor demonstrates that mTORC1 controls PNS myelination through the mTORC1–RXRγ–SREBP–lipid biosynthesis axis; mTORC1 loss results in delayed myelination, hypomyelination, aberrant lipid composition, and reduced nerve conduction velocity [104].

The relationship between mTORC1 and postinjury remyelination is more nuanced than a simple linear activator model. Genetic disruption of key inhibitory components upstream of mTORC1 in developing SCs—TSC1 or PTEN—paradoxically causes apparent delays in developmental myelination and postinjury remyelination despite hyperactivation prior to the myelinating phase [94, 105, 106]. This biphasic, context‐dependent role—inhibitory before myelination onset, stimulatory afterward—has direct implications for the design of therapeutic strategies targeting mTORC1 during remyelination.

### 5.3 Epigenetic Regulation: PRC2, HDACs, and Chromatin Remodeling

Epigenetic regulation constitutes a critical and increasingly well‐characterized control layer of SC myelination and remyelination. The PRC2, via histone H3K27 trimethylation, maintains transcriptional silencing of antimyelinating genes in mature SCs. The PRC2 component EED catalyzes H3K27 methylation and suppresses key nerve repair genes including Shh, Gdnf, and Bdnf; their activation following injury is accompanied by loss of H3K27 methylation. Analysis of SC‐specific Eed‐null mice after nerve injury demonstrates that reversal of Polycomb repression is a rate‐limiting step for transcription of Nrg1 Type I, necessary for effective remyelination, while Eed loss also causes impaired SC proliferation postinjury and significant induction of Cdkn2a cell cycle inhibitor genes [107].

HDAC1 and HDAC2 act through complementary mechanisms during the remyelination phase: downstream of NRG1/ErbB signaling, they suppress the repair SC program by deacetylating c‐JUN target gene promoters, enabling KROX20 upregulation and re‐engagement of compact myelin gene transcription [108]. The NuRD chromatin remodeling complex, in which CHD4 interacts with NAB1/2 core repressors required by EGR2, provides the enzymatic mechanism for HDAC‐dependent silencing during both developmental myelination and remyelination [44].

### 5.4 Noncoding RNA in SC Myelination and Remyelination

Noncoding RNAs constitute a growing and functionally important regulatory layer in SC myelination [109]. Among microRNAs, miR‐138 targets the 3′‐UTR of Sox2 mRNA, and its upregulation during myelination contributes to suppression of the immature SC state. miR‐9 promotes SC proliferation while suppressing differentiation by targeting EGR2/KROX20; miR‐29 family members regulate SC apoptosis and survival pathways. Several miRNAs are dynamically regulated after nerve injury: miR‐21 is strongly upregulated in repair SCs and promotes macrophage‐like phagocytic function; miR‐34a modulates the balance between SC autophagy and apoptosis; miR‐222 promotes SC proliferation early after injury.

In the lncRNA category, the EGR2 promoter antisense RNA (EGR2‐AS) recruits PRC2 components to the EGR2 promoter while recruiting histone activating marks to c‐JUN target gene promoters, orchestrating epigenetic switching between myelinating and repair SC states [110, 111, 112]. Circular RNAs expressed in SCs after nerve injury have been identified through RNA‐seq studies, with several injury‐regulated circRNAs showing binding sites for SC‐relevant miRNAs, though their mechanistic roles remain to be fully characterized [113].

### 5.5 Lipid Metabolism and Cholesterol in Remyelination

The synthesis of large quantities of myelin during remyelination imposes extraordinary biosynthetic demands on SC lipid metabolism. Cholesterol is the rate‐limiting component: it is required for membrane fluidity and the lipid raft domains where major myelin proteins such as P0 concentrate, and for ER export of P0 itself through the cholesterol recognition/interaction amino acid consensus motif [114]. After injury and demyelination, cholesterol derived from myelin debris is recycled by SCs through a process involving liver X receptors (LXR) and ABCA1/ABCG1 transporters, which mediate cholesterol efflux from myelin‐laden lysosomes to the ER and ultimately to newly formed myelin [115].

SC‐specific deletion of squalene synthase (FDFT1) demonstrates the indispensability of de novo cholesterol synthesis for remyelination even when exogenous cholesterol is available, causing severe and irreversible hypomyelination with failure of myelin compaction [116]. Fatty acid synthesis (FAS) is equally indispensable: SC‐specific deletion of FASN causes severe hypomyelination with reduced compact myelin and delayed maturation of conduction velocity [117]. Synthesis of galactolipids—galactosylceramide and its sulfated derivative sulfatide—is also required, as mice lacking these lipids develop progressive neuropathy with paranodal loop disruption and conduction abnormalities [118]. The coordination of individual myelinating SCs—which must produce >5000 µm^2^ of myelin per day at peak synthesis—is achieved through simultaneous regulation of myelin gene transcription by EGR2/KROX20 and lipid biosynthesis gene transcription by SREBP1/2, with mTORC1 acting as the integrating metabolic sensor [119].

The preceding sections detail the intrinsic programs driving remyelination. Yet their execution is inseparable from the extrinsic microenvironment: the same demyelination necessitating lipid and cholesterol synthesis also generates lipid‐rich debris that, if uncleared, directly inhibits axon regeneration. Resolving this synthesis‐versus‐clearance tension is the task of the intraneural immune system, which is discussed next.

## 6 The Immune Microenvironment in Peripheral Nerve Remyelination

Immune involvement in peripheral nerve remyelination unfolds in sequential phases, beginning with the rapid recruitment and diversification of innate myeloid cells and followed by the slower, more precisely regulated contribution of T lymphocytes.

### 6.1 Innate Immune Priming: Resident Macrophages, Neutrophils, and Mast Cells

The PNS is equipped with a resident innate immune surveillance population—endoneurial macrophages—constituting approximately 2–9% of total endoneurial cells under homeostatic conditions and expanding dramatically following injury [84]. Within the first 24 h of nerve injury, SC‐derived cytokines including LIF, IL‐6, and CCL2 trigger mast cell degranulation and neutrophil infiltration, generating an initial proinflammatory environment that potentiates further immune cell recruitment [120]. Notably, conditional knockout studies have demonstrated that CCL2 is not strictly necessary for macrophage accumulation, myelin clearance, or axon regeneration in the PNS; without CCL2, other CCR2 chemokines including CCL7 and CCL12, resident macrophage proliferation, and CCR2‐independent monocyte recruitment can compensate [120]. This chemokine receptor redundancy provides resilience to the inflammatory response but also complicates single‐target anti‐inflammatory therapeutic strategies.

### 6.2 Macrophage Diversity in Nerve Repair: Beyond the M1/M2 Binary

The classical M1/M2 polarization framework has been a useful heuristic for describing the temporal shift of macrophage function after PNI, but it is now widely recognized—across fibrosis, wound‐healing, and PNI research alike—as an oversimplification of the actual spectrum of macrophage fates. Single‐cell and lineage‐tracing studies in multiple injury contexts show that macrophages occupy a continuum of mixed and transitional states rather than two discrete phenotypes, and that at least one additional, nonpolarization fate—macrophage‐myofibroblast transition (MMT)—contributes directly to nerve repair. The M1‐to‐M2 transition described below should therefore be read as one axis of macrophage behavior in the injured nerve, not as the complete picture.

#### 6.2.1 The M1‐to‐M2 Transition as a Partial Remyelination Gate

In the 3–7 days after injury, circulating monocytes recruited in a CCR2‐dependent manner differentiate into proinflammatory (M1‐like) macrophages that collaborate with SCs to phagocytose myelin debris, secreting TNF‐α, IL‐1β, and reactive oxygen species to amplify local inflammation and accelerate myelin breakdown. As debris clearance proceeds, macrophages transition to an anti‐inflammatory (M2‐like) phenotype characterized by IL‐10 secretion, Arginase‐1 expression, and production of neurotrophic and axon growth‐promoting factors including VEGF‐A and IGF‐1 [121]. The balance and timing of this M1‐to‐M2 transition substantially influences the efficiency of remyelination. IL‐33 administration significantly increased M2 macrophage numbers in injured sciatic nerves without affecting M1 populations, promoting axon regeneration and enhanced remyelination [122]. Conversely, persistent M1 macrophage activation—occurring in diabetic neuropathy or aging—is associated with impaired myelin clearance and delayed remyelination [123, 124, 125, 126].

#### 6.2.2 MMT: A Nonpolarization Fate That Supports Late‐Stage Repair

Beyond shifting along the M1–M2 axis, a subset of macrophages in the injured nerve undergoes a qualitatively different fate—transdifferentiation into myofibroblast‐like cells that coexpress macrophage markers (CD68/F4/80) and the myofibroblast marker α‐smooth muscle actin (α‐SMA), a process termed MMT. MMT was originally described as a source of collagen‐producing, profibrotic cells in kidney, lung, and liver fibrosis, but recent work demonstrates that in PNI, MMT plays a distinct, largely proreparative role: in the late stage of nerve injury, a population of macrophages undergoes MMT and deposits collagen at the injury site, a process that is associated with the elimination of the macrophage population itself and with improved functional regeneration, suggesting that MMT operates as a resolution mechanism that converts surplus late‐stage macrophages into matrix‐producing cells while simultaneously terminating their inflammatory contribution [127]. This finding reframes collagen deposition after PNI—previously attributed largely to resident and infiltrating fibroblasts—as a partly macrophage‐derived process, and it identifies MMT timing and magnitude as a potential regulatory node distinct from, and complementary to, the M1‐to‐M2 axis: whereas M1‐to‐M2 polarization principally governs debris clearance and the early trophic environment, MMT appears to act later, contributing to ECM remodeling of the regeneration track and to the physical resolution of the macrophage infiltrate. Whether MMT and M1‐to‐M2 polarization represent sequential fates of the same macrophage population, parallel fates of distinct subpopulations, or overlapping/mixed states in individual cells remains to be resolved at single‐cell resolution in PNI models specifically, and represents an important open question raised by extending the fibrosis literature to the peripheral nerve.

#### 6.2.3 Extracellular Vesicles and Electrical Stimulation as Convergent Modulators of Macrophage Fate

Electrical stimulation (ES) of injured nerves provides an additional mechanism for controlling macrophage phenotype: ES modulates local SCs to increase exosome release, leading to phenotypic shift from M1 to M2, downregulating proinflammatory cytokines and promoting prorepair factors to accelerate myelin debris clearance and remyelination [128]. SC–macrophage crosstalk mediated by extracellular vesicles represents an emerging paradigm for understanding how multiple therapies converge on the same immunological bottleneck; it remains an open question whether such interventions also modulate the magnitude or timing of MMT alongside classical M1‐to‐M2 polarization [129, 130, 131, 132].

### 6.3 T Lymphocytes and Adaptive Immunity in Peripheral Nerve Remyelination

Beyond the myeloid compartment, the adaptive immune system—principally T lymphocytes—provides a further, more precisely tuned layer of regulation. CD4+ regulatory T cells (Tregs) act in parallel with, rather than in place of, the macrophage‐centered mechanisms above: beyond their canonical immunosuppressive role, Tregs secrete prorepair mediators that act directly on SCs.

Although macrophages are essential during the acute phase of Wallerian degeneration, the transition to sustained remyelination and functional recovery involves a more nuanced, adaptive immune response than macrophage biology alone can explain. In particular, Tregs have emerged as key modulators that act beyond classical inflammation‐resolution, interacting directly with SCs to support the regenerative microenvironment. Beyond their canonical immunosuppressive role, Tregs are increasingly recognized to secrete prorepair mediators that act directly on resident tissue cells across multiple organ systems, a nonimmunosuppressive repair function that is only beginning to be characterized in peripheral nerve [133]. Within the injured nerve specifically, Treg infiltration and local proliferation have been associated with suppression of the Th1 response and with a more favorable, less inflammatory microenvironment for axon regrowth, positioning Tregs as a regulatory layer that operates in parallel with, rather than in place of, the macrophage‐centered mechanisms described above. Together with the M1‐to‐M2 axis and MMT, Treg–SC interaction underscores that remyelination after PNI is governed by a multicellular immune network rather than by macrophage polarization state alone, and future work integrating single‐cell profiling of macrophages, myofibroblast‐transitioned cells, and T‐cell subsets within the same injury timeline will be needed to resolve how these programs are coordinated.

T lymphocytes contribute to peripheral nerve repair through both beneficial and pathological mechanisms. CD4+ Tregs are recruited to injured nerves and exert pleiotropic proregenerative effects. Tregs suppress neuropathic pain by inhibiting CD4+ Th1 responses at the injury site and reducing neuroinflammation at sensory ganglia through IL‐10 signaling [134]. Beyond immune suppression, Tregs interact with SCs through amphiregulin (AREG), a low‐affinity EGF receptor ligand that promotes myelination and supports SC proliferation. AREG is upregulated in SCs after sciatic nerve injury and stimulates SC proliferation and migration through ERK1/2 cascade activation [135, 136, 137].

Notably, aging impairs the proregenerative function of Tregs in parallel with aging‐associated decline in SC repair capacity. Studies in CNS remyelination directly demonstrate that aged Tregs lose the ability to support remyelination, an effect restorable by young systemic environments, identifying immune rejuvenation as a potential strategy to overcome aging‐associated remyelination failure [138]. Whether the same Treg aging mechanism operates in peripheral nerve remyelination remains an open question of clinical importance. CD8+ cytotoxic T cells have been implicated in axonal injury in autoimmune neuropathies such as Guillain–Barré syndrome (GBS), where complement‐mediated attack on nodal and paranodal proteins disrupts saltatory conduction [139, 140, 141] (Table 2).

**TABLE 2:** Immune cell populations and their temporal roles in peripheral nerve remyelination.

Immune population	Temporal window	Key markers	Principal mechanism	Net effect on remyelination	References
Resident endoneurial macrophages	Homeostatic; expands acutely postinjury	—	Rapid local expansion, early debris sampling	Innate priming	[84]
Mast cells and neutrophils	0–24 h	—	SC‐derived LIF/IL‐6/CCL2‐triggered degranulation and infiltration	Initiates inflammatory milieu	[120]
M1‐like macrophages	Day 3–7	iNOS+/CD86+	TNF‐α, IL‐1β, ROS‐mediated phagocytosis of myelin debris	Debris clearance (harmful if persistent)	[121]
M2‐like macrophages	∼Day 7 onward	Arg1+/IL‐10+	VEGF‐A, IGF‐1 secretion; IL‐33‐inducible	Proremyelination	[121, 122]
MMT cells	Late stage	CD68/F4/80+ α‐SMA+	Collagen deposition; self‐elimination of macrophage pool	ECM remodeling/resolution	[127]
Tregs	∼Day 7 onward	CD4+CD25+FoxP3+	IL‐10 (Th1 suppression); AREG→ERK1/2 signaling on SCs	Prorepair, direct SC support	[133, 134, 135]
Aged Tregs	Aging context	—	Loss of proregenerative function; restorable by young systemic milieu	Contributes to age‐related remyelination failure	[138]
CD8+ cytotoxic T cells	Autoimmune context (e.g., GBS)	CD8+	Complement‐mediated attack on nodal/paranodal proteins	Antiremyelination (pathological)	[139]

Abbreviations: AREG, amphiregulin; Arg1, Arginase 1; CCL2, C–C motif chemokine ligand 2; CD4, cluster of differentiation 4; CD8, cluster of differentiation 8; CD25, cluster of differentiation 25; CD68, cluster of differentiation 68; CD86, cluster of differentiation 86; ECM, extracellular matrix; ERK1/2, extracellular signal‐regulated kinase 1/2; F4/80, EGF‐like module‐containing mucin‐like hormone receptor‐like 1 (macrophage marker); FoxP3, forkhead box P3; GBS, Guillain–Barré syndrome; IGF‐1, insulin‐like growth factor‐1; IL‐1β, interleukin‐1 beta; IL‐6, interleukin‐6; IL‐10, interleukin‐10; IL‐33, interleukin‐33; iNOS, inducible nitric oxide synthase; LIF, leukemia inhibitory factor; MMT, macrophage‐to‐myofibroblast transition; ROS, reactive oxygen species; SC, Schwann cell; TNF‐α, tumor necrosis factor alpha; Treg, regulatory T cell; VEGF‐A, vascular endothelial growth factor A; α‐SMA, alpha‐smooth muscle actin.

Together, the innate and adaptive arms of the intraneural immune system constitute a temporally layered network licensing the remyelination program financed by lipid and cholesterol biosynthesis (Figure 3). This network is not uniformly robust: aging, metabolic disease, and mechanical or genetic insult can each disrupt one or more components.

**FIGURE 3:**
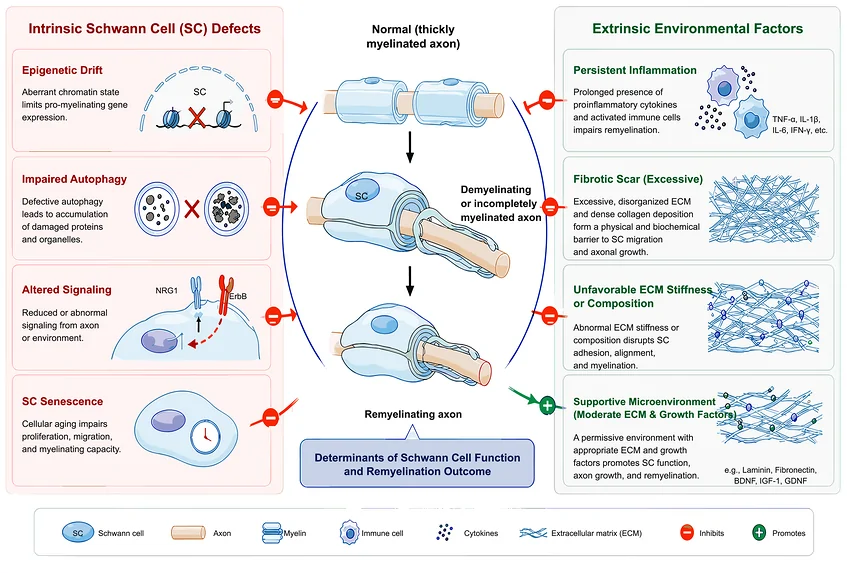
Coordination of metabolic, innate, and adaptive immune programs during peripheral nerve remyelination. (A) The metabolic pillar: SREBP2/mTORC1‐driven lipid and cholesterol biosynthesis supplies membrane for new compact myelin but generates lipid‐rich debris. (B) The innate immune pillar: resident macrophages, mast cells, and neutrophils prime the response; monocyte‐derived M1‐like macrophages clear debris before transitioning to M2‐like and MMT states. (C) The adaptive immune pillar: Tregs act on Schwann cells via AREG signaling and suppress Th1 responses, while CD8+ T cells mediate pathological demyelination in autoimmune neuropathies. Bottom: approximate temporal overlap of the three pillars after injury. Abbreviations: AREG, amphiregulin; CD8, cluster of differentiation 8; ECM, extracellular matrix; ERK1/2, extracellular signal‐regulated kinase 1/2; GBS, Guillain–Barré syndrome; IGF‐1, insulin‐like growth factor‐1; IL‐10, interleukin‐10; M1/M2, classically activated (M1)/alternatively activated (M2) macrophage phenotype; MMT, macrophage‐to‐myofibroblast transition; mTORC1, mechanistic target of rapamycin complex 1; SC, Schwann cell; SREBP2, sterol regulatory element‐binding protein 2; Th1, T helper Type 1 cell; Treg, regulatory T cell; VEGF‐A, vascular endothelial growth factor A.

## 7 Remyelination Failure: Aging, Disease, and Molecular Barriers

### 7.1 SC Senescence as a Core Mechanism of Age‐Related Remyelination Failure

While peripheral nerve remyelination is more robust than CNS remyelination, its efficiency declines with age. The most significant recent advance has been the discovery that repair SCs transition to a senescent phenotype during aging and chronic denervation, characterized by reduced c‐JUN expression and secretion of axonal regeneration inhibitory factors. Elimination of senescent SCs via systemic senolytic drug treatment or genetic targeting improved nerve regeneration and functional recovery and increased c‐JUN expression in both aging and chronic denervation conditions [142, 143, 144, 145, 146]. p16‐positive senescent cell nuclei are present in up to 60% of SOX10‐positive cells in aged or chronically denervated nerves, a remarkable prevalence that explains why the repair capacity of aged nerves is fundamentally compromised rather than merely slowed.

The molecular basis of SC senescence involves DNA damage response activation arising from the high replicative stress experienced by repair SCs after nerve injury. Aged SCs cannot efficiently secrete GDNF, BDNF, NGF, CNTF, NT‐3, and β‐neuregulin, which are critical for motor neuron survival, neurite growth, myelination, and neuromuscular junction remodeling [147]. Senolytic drugs that selectively eliminate p16‐positive senescent cells—including BCL‐2 family inhibitor ABT‐263 (navitoclax) and the quercetin‐dasatinib combination—have been shown to improve axon regeneration in aged mice by restoring the regeneration‐permissive environment of the distal nerve stump [148].

### 7.2 Metabolic Disease: Diabetic and Chemotherapy‐Induced Neuropathies

In diabetic peripheral neuropathy (DPN), chronic hyperglycemia disrupts SC mitochondrial function, promotes oxidative stress through advanced glycation end products and reactive oxygen species, impairs autophagy and myelin clearance, and reduces NRG1/ErbB2 signaling. DPN is characterized by a dying‐back axonopathy with secondary demyelination and severely impaired remyelination; in animal models, insulin replacement or antioxidant treatment partially reverses these abnormalities [149]. The NMNAT2–SARM1‐mediated axon degeneration cascade is implicated in diabetic neuropathy, with SARM1‐null mice showing protection in DPN models, suggesting that targeting the NMNAT2–SARM1‐mediated axon degeneration cascade may benefit metabolic neuropathies [83, 150, 151].

Chemotherapy‐induced peripheral neuropathy caused by paclitaxel, cisplatin, vincristine, and bortezomib involves direct SC toxicity, axonal transport disruption, and mitochondrial dysfunction. Perioperative ES accelerates axon growth, remyelination of regenerated axons, and terminal organ reinnervation even when surgical intervention is delayed; several recent studies confirm ES as a clinically translatable technique enhancing functional recovery in PNI patients requiring surgical treatment [152, 153, 154].

### 7.3 Compressive and Hereditary Neuropathies

Chronic compressive neuropathies cause focal paranodal demyelination followed by remyelination, and if compression persists, eventually axonal degeneration. Paranodal junctions formed by the Caspr–contactin–NF155 tripartite complex are particularly vulnerable to mechanical deformation and are among the earliest structures disrupted in compressive neuropathies. Postdecompression remyelination is generally effective for mild‐to‐moderate cases but may be incomplete after severe or prolonged compression [155, 156, 157].

Hereditary demyelinating neuropathies including CMT1A (PMP22 duplication) and CMT1B (MPZ mutation) represent conditions of chronic impaired myelination where therapeutic enhancement of myelination offers genuine clinical benefit. The discovery that onion bulb formation in CMT1A involves NRG1 Type I autocrine signaling in SCs, driving a continuous futile demyelination–remyelination cycle, identifies soluble NRG1 as a paradoxical disease driver in hereditary neuropathy and proposes SC‐specific MEK inhibition as a strategy to break this pathological cycle [103] (Figure 4). The barriers surveyed above share a common implication: each identifies a druggable node within the programs detailed. The focus next turns from mechanism to intervention.

**FIGURE 4:**
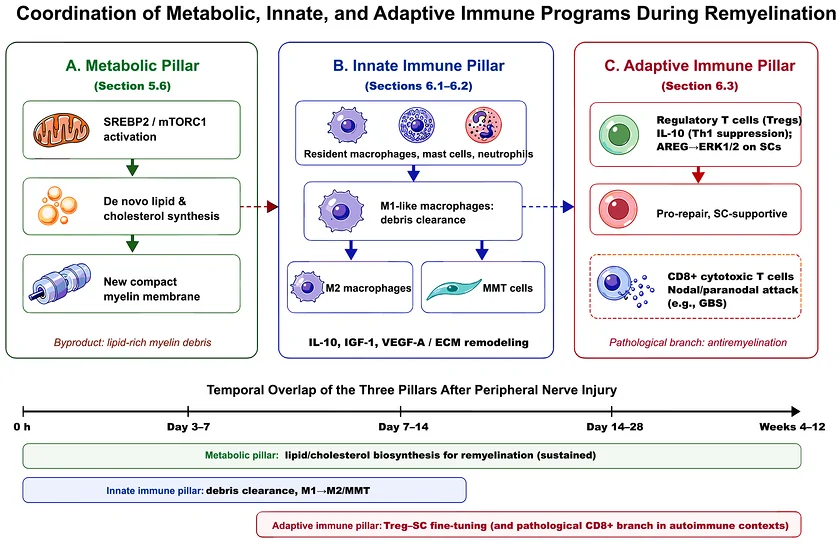
Intrinsic and extrinsic determinants of peripheral nerve remyelination. Schematic overview of the major intrinsic Schwann cell defects and extrinsic microenvironmental factors that regulate peripheral nerve remyelination. Intrinsic determinants include epigenetic drift, impaired autophagy, cellular senescence, and dysregulated NRG1/ErbB signaling, all of which reduce Schwann cell plasticity and myelinating capacity. Extrinsic factors include persistent inflammation, abnormal ECM remodeling, excessive fibrotic scar formation, and impaired trophic support. In contrast, a permissive ECM with appropriate collagen organization and growth factor availability supports Schwann cell migration, axonal regeneration, and remyelination. The central panel illustrates the transition from normal myelination to demyelination and subsequent remyelination by Schwann cells. Potential therapeutic intervention points targeting both intrinsic and extrinsic mechanisms are indicated. Abbreviations: ECM, extracellular matrix; ErbB, erythroblastosis oncogene B receptor; NRG1, Neuregulin‐1; SC, Schwann cell.

## 8 Therapeutic Strategies for Enhancing Myelination and Remyelination

### 8.1 Biological Strategies: Growth Factors and Cellular Therapies

Current and emerging therapeutic approaches for enhancing peripheral nerve remyelination can be broadly categorized into biological, biomaterial, biophysical, and pharmacological strategies (Table 3). The following sections detail the underlying mechanisms and evidence supporting each.

**TABLE 3:** Preclinical and clinical therapeutic strategies targeting peripheral nerve remyelination.

Strategy	Target/mechanism	Model	Key outcome	Clinical stage	References
Recombinant NRG1‐β1	ErbB2/ErbB3 → mTORC1	Rat sciatic crush	↑ myelin thickness, ↑ conduction velocity	Preclinical	[158]
CNTF gene therapy (AAV)	GFRα1/RET → ERK/AKT	Rat transection + conduit	↑ axon count, ↑ remyelination	Preclinical	[159, 160, 161]
Autologous SC transplant	Direct myelination; trophic support	Human SCI/PNI trials	Safe; moderate improvement	Phase I/II	[162]
iPSC‐derived SCs	Remyelination + paracrine	Rat sciatic crush	↑ g‐ratio, ↑ functional recovery	Preclinical	[163, 164, 165]
PLGA/collagen conduit + NRG1	Structural bridge + SC recruitment	Rat 15 mm gap	Comparable to autograft (short gap)	Preclinical/clinical CE‐marked	[166]
ES (1 h, 20 Hz)	BDNF/cAMP; accelerate crossing	Human femoral nerve repair	↑ reinnervation speed, ↑ strength	Phase II/III	[167]
Ascorbic acid	Collagen synthesis; SC myelination	CMT1A mouse	↑ myelin thickness	Preclinical/Phase II trials	[168]
Valproic acid (HDACi)	HDAC1/2; ↑ KROX20	Rat sciatic crush	↑ myelination; ↑ recovery	Preclinical	[169]
Navitoclax/dasatinib + quercetin (senolytics)	Senescent SC elimination; restore c‐JUN	Aged mouse sciatic crush	↑ axon regeneration; ↑ c‐JUN; ↑ functional recovery	Preclinical (Phase I/II for aging indications)	[170]

Abbreviations: AAV, adeno‐associated virus; AKT, protein kinase B; BDNF, brain‐derived neurotrophic factor; cAMP, cyclic adenosine monophosphate; CMT1A, Charcot–Marie–Tooth disease Type 1A; CNTF, ciliary neurotrophic factor; ErbB2/ErbB3, Erb‐B2 receptor tyrosine kinase 2/3; ERK, extracellular signal‐regulated kinase; ES, electrical stimulation; GFRα1, GDNF family receptor alpha 1; HDACi, histone deacetylase inhibitor; iPSC, induced pluripotent stem cell; mTORC1, mechanistic target of rapamycin complex 1; NRG1‐β1, Neuregulin‐1 beta1; PLGA, poly(lactic‐co‐glycolic acid); PNI, peripheral nerve injury; RET, RET proto‐oncogene (rearranged during transfection); SC, Schwann cell; SCI, spinal cord injury.

#### 8.1.1 Neuregulin‐1‐Based and Growth Factor Approaches

The central role of NRG1 Type III in SC myelination makes it a primary therapeutic target for PNI. Intramuscular injection or delivery via slow‐release biomaterials of recombinant NRG1 (EGF‐like domain, NRG1‐β1) following sciatic nerve injury consistently accelerates remyelination, increases myelin thickness, and improves functional recovery [171, 172]. Mechanistically, NRG1‐β1 activates ErbB2/ErbB3→PI3K–AKT→mTORC1 in SCs to promote SC survival and proliferation and suppresses the c‐JUN‐mediated repair SC program [172]. An important nuance is the subtype‐specificity of NRG1 signaling: while transmembrane NRG1 expressed on the axon surface signals promyelination, soluble NRG1 isoforms expressed by SCs immediately after injury suppress myelination genes and promote SC survival and dedifferentiation [173]. Therapeutic strategies must therefore target transmembrane rather than soluble NRG1 to achieve promyelination outcomes.

Other growth factors promoting SC myelination include GDNF, BDNF, CNTF, and IGF‐1, each converging on KROX20 activation. Combined delivery of NGF and GDNF via PLGA microspheres within nerve guidance conduits (NGCs) demonstrates dose‐dependent, synergistic enhancement of myelination in preclinical models, supporting combinatorial approaches exploiting nonredundant mechanisms [174, 175, 176]. Sustained controlled‐release biomaterial systems—including hydrogels, aligned nanofiber scaffolds with growth factor‐loaded microspheres, and AAV‐mediated gene therapy—address the fundamental challenge of short in vivo half‐life of recombinant proteins.

#### 8.1.2 SC Transplantation and iPSC‐Derived Glial Cells

Autologous SC transplantation remains one of the most promising cell‐based strategies for enhancing peripheral nerve remyelination. SCs harvested from sural nerve biopsies, expanded in vitro using mitogenic cocktails (heregulin, forskolin, PDGF, bFGF), and transplanted directly into nerve conduits or injury sites integrate into the endogenous SC population, remyelinate regenerating axons, and provide sustained paracrine trophic support. The Miami Project to Cure Paralysis has advanced autologous SC transplantation to Phase I/II clinical trials for spinal cord injury, with encouraging safety data and evidence for modest functional improvement. Advances in purification and expansion protocols have shortened ex vivo expansion time while preserving myelinating capacity [177].

Human iPSC‐derived SC‐like cells provide a scalable allogeneic alternative. Recent differentiation protocols achieve robust SC phenotypes within 20–30 days through sequential application of BMP inhibitors, NRG1, and neuregulin receptor activation, producing cells expressing S100β, SOX10, and p75NTR that myelinate neurons in vitro and enhance remyelination in rodent PNI models [178]. Remaining translational challenges include immune compatibility in allogeneic applications, manufacturing scalability for clinical‐grade production, and safety concerns from residual undifferentiated pluripotent cells requiring stringent quality control.

### 8.2 Biomaterial Strategies: NGCs and Tissue‐Engineered Bands of Büngner

Bridging nerve gaps exceeding 3–5 mm requires a structural scaffold to guide SC migration and axon regeneration. Biomaterial NGCs fabricated from collagen, chitosan, PLGA, or polyurethane can be loaded with growth factors, ECM components (laminin, fibronectin), and aligned nanofibers guiding SC migration and axon extension [179]. Advanced conduit designs incorporate conductivity (polypyrrole, carbon nanotubes), internal microchannels replicating fascicular organization, and controlled‐release microspheres for sustained growth factor delivery [180]. Decellularized nerve allografts preserve native intraneural architecture and basement membrane, providing an excellent substrate for SC repopulation and remyelination.

A particularly innovative development is the engineering of tissue‐engineered Bands of Büngner (TE BoBs)—prefabricated, implantable SC‐laden microtissues mimicking the anisotropic structure and function of naturally occurring Büngner bands. The extent of axon regeneration decreases sharply with increasing gap length because host SCs generally cannot penetrate large acellular scaffolds; TE BoBs address this limitation by preseeding aligned SC structures within a biomaterial framework, enabling rapid axon acceleration after implantation [181].

### 8.3 Biophysical Strategies: ES

Brief intraoperative ES of the proximal nerve stump immediately following surgical repair has garnered support from multiple prospective randomized controlled trials (RCTs), making it the most advanced clinical strategy for enhancing peripheral nerve regeneration. Basic and clinical work demonstrated that 1‐h ES (20 Hz, continuous) following femoral nerve repair significantly accelerated nerve regeneration and improved functional motor recovery compared with sham stimulation [182]; subsequent RCTs have confirmed efficacy across different injury types and anatomical locations [183].

The mechanisms by which ES promotes remyelination—distinct from its effects on axon regeneration speed—have been elucidated in important recent studies. ES increased the number of myelinated fibers and myelin thickness at 4 and 8 weeks postinjury; P0 levels were elevated in ES‐treated groups both in vitro and in vivo; early peaking of Par‐3 in ES‐treated groups indicated earlier onset of SC myelination; and the therapeutic effect on myelination was mediated through enhanced BDNF signaling [184]. ES also increases neuronal intracellular cyclic AMP through retrograde action potentials and induces more rapid upregulation of regeneration‐associated genes; delayed ES delivery, even at 4–24 weeks postinjury, still enhances axon regeneration, remyelination, and motor functional recovery, extending its clinical applicability beyond intraoperative use [185, 186].

### 8.4 Pharmacological Strategies: mTORC1, Senolytics, and Beyond

Translating mechanistic insight into effective pharmacological therapy for peripheral nerve demyelination and remyelination has proven more difficult than the preclinical literature initially suggested. The subsections below critically examine four of the most actively pursued targets—mTORC1 signaling, cholesterol/lipid metabolism, ascorbic acid, and cellular senescence—placing each preclinical success alongside its clinical translation record, since a therapeutic target with strong mouse‐model rationale but no positive human trial is scientifically distinct from one already validated in patients.

#### 8.4.1 mTORC1: A Temporally Biphasic and Context‐Dependent Target

Rapamycin (mTORC1 inhibitor) paradoxically impairs chronic remyelination while promoting early myelin debris clearance, indicating the need for temporally precise drug delivery strategies [94]. This apparent paradox has a mechanistic explanation: mTORC1 activity is normally low in mature, myelinating SCs but is transiently and robustly reactivated in dedifferentiating SCs immediately after injury, where it drives c‐JUN translation and thereby licenses myelin clearance; genetic ablation of mTORC1 signaling at this early stage delays demyelination and, indirectly, subsequent remyelination [187]. Conversely, sustained mTORC1 activity is required later for the anabolic, membrane‐synthetic demands of remyelination itself, and its effects are dose‐ and context‐dependent rather than uniformly beneficial or harmful across the SC lineage [188]. This biphasic requirement explains why systemic, temporally unconstrained mTORC1 inhibition (or activation) produces inconsistent preclinical results, and it has motivated a shift toward pathway‐selective or temporally restricted dosing strategies. A recent example is the evaluation of PI3K/Akt/mTOR pathway modulators in rodent models of PMP22 gene‐dosage neuropathies, which found stage‐dependent efficacy consistent with the biphasic model above and underscores that clinical translation will likely require injury‐phase‐specific rather than continuous dosing regimens [189].

#### 8.4.2 Cholesterol and Lipid Metabolism: A Mechanistically Compelling but Clinically Untested Target

Cholesterol is not a passive structural lipid but a rate‐limiting determinant of myelin assembly, which makes myelin lipid metabolism an intuitively attractive pharmacological target—yet it remains the least clinically developed of the strategies discussed in this section. The strongest proof‐of‐concept comes not from cholesterol itself but from broader myelin lipid supplementation: in a rat model of CMT1A, myelinating SCs show a developmental deficit in the transcription of lipid‐biosynthetic genes, leading to a distorted myelin protein‐to‐lipid stoichiometry; dietary supplementation with phosphatidylcholine and phosphatidylethanolamine—precursors that feed into the same biosynthetic network as cholesterol—was sufficient to restore the number of myelinated axons and substantially ameliorate the neuropathic phenotype [190]. This result is mechanistically important for two reasons directly relevant to therapeutic strategy: first, it demonstrates that a dietary/nutritional intervention, rather than a receptor‐targeted drug, can correct a lipid‐synthesis deficit in vivo; second, it argues against a naïve “cholesterol‐lowering” approach—because SCs synthesize nearly all of their myelin cholesterol autonomously and cannot efficiently import it from the circulation, systemic cholesterol‐lowering agents such as statins are mechanistically more likely to exacerbate than to relieve a demyelinating phenotype, and this caution has been borne out by case reports linking statin therapy to worsening of pre‐existing CMT neuropathy rather than by any positive statin trial in remyelination. Upstream of lipid supplementation, the transcriptional coupling between cholesterol biosynthesis and myelin gene expression and the identification of additional transcriptional regulators of SC cholesterol biosynthesis, such as the RNA‐binding protein Qki acting through Srebp2, illustrate that the field currently has more validated upstream regulators of cholesterol synthesis than downstream pharmacological levers [191]. No cholesterol‐supplementation or cholesterol‐synthesis‐inducing compound has yet entered human trials for peripheral demyelinating disease, so at present this target should be regarded as biologically well justified but clinically unproven—a gap that dietary lipid‐precursor supplementation strategies, rather than direct cholesterol administration, seem best positioned to close first.

#### 8.4.3 Ascorbic Acid: A Cautionary Tale in Translation

Ascorbic acid increases SC basement membrane collagen synthesis and promotes myelination in culture and, in the C22 transgenic mouse model of CMT1A, high‐dose oral ascorbic acid substantially ameliorated the neuropathic phenotype and reduced PMP22 overexpression, prompting considerable early enthusiasm because ascorbic acid was already an United States Food and Drug Administration (US FDA)‐approved, low‐risk compound [168]. This preclinical result, however, did not translate: three independent 1‐year Phase II trials in children and adults with CMT1A, followed by a 2‐year, 271‐patient, multicenter, placebo‐controlled trial (CMT‐TRIAAL/CMT‐TRAUK), all failed to show a significant benefit of ascorbic acid on neuropathy severity or nerve conduction [192], and a subsequent Cochrane systematic review concluded that the cumulative trial evidence does not support ascorbic acid as a treatment for CMT1A [193]. The ascorbic acid experience is instructive for the field as a whole: a mechanistically plausible, single‐target intervention with a robust rodent phenotype produced essentially no effect across more than 500 treated patients in aggregate, most plausibly because rodent PMP22‐overexpression models do not fully recapitulate the chronicity, fibre‐type heterogeneity, and secondary axonal loss of established human CMT1A, and because oral bioavailability and achievable peripheral nerve concentrations of ascorbic acid in adult patients may be substantially lower than in juvenile rodents. This case argues for caution in extrapolating single‐compound rodent efficacy directly to clinical benefit and for prioritizing combination or stage‐specific strategies over monotherapies validated only in short‐duration animal models.

#### 8.4.4 HDAC Inhibitors

HDAC inhibitors such as glycyrrhizic acid and valproic acid may enhance SC differentiation and myelination by enhancing HDAC1/2‐dependent silencing of inhibitory transcription factors including c‐JUN and ID2 [194]. As with mTORC1, this mechanism is double‐edged: because HDAC1/2 activity and NuRD‐complex function are also required for normal developmental myelination and for injury‐induced dedifferentiation, pharmacological HDAC inhibition carries a theoretical risk of suppressing the very repair‐state transition it is intended to support if applied without regard to injury stage, a consideration that has not yet been systematically addressed in peripheral nerve models and represents an open question for the field.

#### 8.4.5 Senolytics: Clearing the Senescent Burden

Senolytic targeting of senescent SCs is an emerging strategy with direct preclinical validation. Repair SCs generated after PNI can themselves transition into a senescent, p16^INK4a^/p21^CIP1^‐positive state characterized by diminished c‐JUN expression and a senescence‐associated secretory phenotype (SASP) that actively inhibits axonal regrowth, and this transition is markedly more prominent in aged animals and after chronic denervation. Systemic elimination of these senescent SCs—either pharmacologically with the BCL‐2/BCL‐xL inhibitor navitoclax (ABT‐263) or the dasatinib‐plus‐quercetin combination, or genetically—restores c‐JUN expression, reduces the inhibitory SASP, and improves axonal regeneration and functional recovery in both aged and chronically denervated nerves [170]. This approach is particularly attractive because navitoclax and dasatinib/quercetin are already in clinical development or approved for other indications (chronic lymphocytic leukemia and oncology, respectively), providing a potentially accelerated regulatory pathway for repurposing in peripheral neuropathy; however, unlike ascorbic acid, senolytics have not yet been tested in any peripheral‐nerve clinical trial, so their translational reliability remains formally untested despite the mechanistic and preclinical rationale being considerably stronger and more recent than that available for ascorbic acid at the time its trials were launched.

#### 8.4.6 Synthesis: What the Comparative Record Teaches

Taken together, these four targets illustrate a graded translational hierarchy rather than a uniform field of “promising therapies.” Ascorbic acid represents the clearest negative result: strong single‐target rodent efficacy that did not survive controlled human trials [192, 193], most likely reflecting species‐ and chronicity‐related limitations of the preclinical model rather than a flaw in the ascorbic acid mechanism itself. mTORC1 and HDAC inhibition occupy an intermediate position: both are mechanistically double‐edged, biphasic, or context‐dependent in animal models, and neither has yet been tested clinically with dosing regimens that respect this complexity, making failure or success in human trials difficult to predict from the current preclinical literature [108, 187, 188]. Cholesterol/lipid metabolism is the most mechanistically compelling target discussed in this review—directly supported by human genetics and by a successful dietary‐supplementation rescue in a rodent CMT1A model [189, 190]—but remains furthest from clinical testing, and the field currently understands the transcriptional upstream regulators of cholesterol biosynthesis better than it understands how to pharmacologically titrate cholesterol delivery to SCs in vivo. Senolytics currently offer the most favorable combination of strong, recent mechanistic rationale and an accelerated regulatory pathway via drug repurposing [142, 145, 146], but their apparent promise remains preclinical and should be interpreted with the ascorbic acid precedent in mind rather than assumed to guarantee clinical success. It is suggested that future pharmacological studies in this field explicitly report the injury stage, dosing window, and species/chronicity limitations of their models, so that the risk of a second ascorbic‐acid‐type translational failure can be assessed prospectively rather than retrospectively. Taken together, these strategies remain at markedly different stages of translational maturity. These observations are synthesized into a forward‐looking assessment of clinical translation and priority directions.

## 9 Clinical Translation

Bridging the preclinical strategies discussed above to actual patient care requires more than mechanistic promise: it requires an accessible clinical product, a regulatory pathway, and, increasingly, biomarkers capable of demonstrating benefit within trial timescales that are feasible for a chronic, slowly regenerating tissue. Recent developments show that these requirements are being met unevenly across the biological, biophysical, and pharmacological strategies.

Among biomaterial‐based approaches, translation has been most successful. In December 2025, the US FDA approved a Biologics License Application for Avance (acellular nerve allograft‐arwx), making it the first processed nerve allograft to receive full biologic licensure, for sensory nerve discontinuities ≤25 mm, based on a randomized, double‐blind Phase III trial demonstrating noninferiority to a collagen nerve cuff [195]. The agency simultaneously granted accelerated approval for larger sensory gaps and for motor and mixed nerve discontinuities on the basis of a surrogate endpoint reasonably likely to predict clinical benefit, with confirmatory trials required to verify durability—an implicit acknowledgment that gold‐standard functional endpoints in nerve‐repair trials take years to mature, and that surrogate‐endpoint pathways may be necessary to keep pace with the underlying biology.

ES illustrates a translation bottleneck of a different kind: not biological uncertainty but device access. Although the 1‐h, 20 Hz postoperative ES protocol has been validated across several RCTs, its adoption was limited for years by the absence of a purpose‐built, easy‐to‐use clinical stimulator. A 2025 pilot study of a single‐use perioperative neurostimulator (PeriPulse) in acute digital nerve transection reported improved sensory thresholds at 6 months in most treated patients relative to historical controls, and established feasibility for a larger confirmatory RCT [196]. ES may therefore be the strategy closest to routine clinical uptake, contingent less on further mechanistic work than on device standardization and reimbursement.

Pharmacological targeting of the NMNAT2–SARM1‐mediated axon degeneration cascade offers a cautionary counterpoint. Several companies have advanced small‐molecule SARM1 inhibitors into early clinical trials for axonal neuropathies, including Lilly's LY3873862 and Nura Bio's NB‐4746. However, mechanistic studies published in 2025 showed that first‐generation active‐site (orthosteric) SARM1 inhibitors can, at subinhibitory concentrations under conditions of mild endogenous SARM1 activation, paradoxically stabilize the enzyme's active conformation and worsen neurodegeneration rather than prevent it [197, 198]. This class‐specific liability—invisible in healthy‐volunteer trials but potentially unmasked in patients with ongoing axonal stress—has led several developers to deprioritize orthosteric inhibitors in favor of allosteric small molecules, gene therapy, and antisense oligonucleotides that suppress SARM1 expression rather than modulating its active site [199]. The episode underscores a theme recurring throughout this review: mechanistic validation in animal models is necessary but not sufficient for clinical translation, and target‐specific toxicity can only be discovered once a therapy reaches the patient population it was designed to treat.

Cell‐ and senescence‐targeted strategies remain the earliest‐stage of the four. Eliminating senescent SCs restores regenerative capacity in aged and chronically denervated rodents, and partial cellular reprogramming has recently been shown to reverse SC senescence in aged rats by restoring stress‐granule homeostasis [200]. Yet no senolytic or reprogramming agent has entered a dedicated peripheral‐nerve clinical trial, and autologous or iPSC‐derived SC transplantation remains confined to small Phase I/II safety cohorts.

Taken together, these four translational tracks—biomaterials furthest advanced via a surrogate‐endpoint regulatory pathway, ES limited by device engineering rather than biology, small‐molecule axon‐protective agents newly confronted with class‐specific safety signals, and cell/senescence‐targeted therapies still preclinical—show that “clinical translation” in peripheral nerve remyelination is not one pipeline but several, each maturing on its own timescale and requiring distinct evidence before reaching patients.

## 10 Future Perspectives and Conclusion

The confluence of single‐cell transcriptomics, gene therapy, and advanced biomaterials positions the field of peripheral nerve remyelination at an inflection point. Six convergent frontier areas are identified that will define the coming decade. First, single‐cell and spatial multiomics have identified at least four functionally distinct SC subpopulations—myelinating, nonmyelinating, immature, and repair—whose proportions and intercellular communication networks change dynamically in injury and disease [201]. Therapeutic strategy design must be cell‐state specific rather than treating SCs as a homogeneous population. Second, AAV‐based gene therapy is rapidly approaching clinical translation. AAV‐mediated expression of dominant‐negative SARM1 constructs in adult mice achieves axon protection after sciatic nerve transection comparable to germline SARM1 knockout, and antisense oligonucleotides targeting SARM1 are progressing toward clinical evaluation in chemotherapy‐induced and diabetic neuropathy [202]. Third, senolytic and regenerative therapies represent a clinically accessible avenue given that senolytics are already in clinical development for aging‐related diseases. The discovery that SC senescence is a therapeutically reversible barrier to remyelination in aged populations [142] provides immediate translational opportunity. Fourth, organ‐on‐chip platforms recreating the complete myelination–demyelination–remyelination cycle in 3D microfluidic environments [203] provide a high‐fidelity preclinical screening bridge between molecular discovery and in vivo validation that is sorely needed given the poor predictive value of current rodent models for human outcomes. Fifth, smart electroactive biomaterials that combine structural guidance, electrical conductivity, and controlled growth factor release represent the next generation of NGCs capable of simultaneously addressing multiple barriers to remyelination. Sixth, and most transformatively, rationally sequenced combination precision therapies—concurrent early ES to accelerate axon‐SC contact, senolytic clearance of the distal stump, sustained NRG1 delivery via smart biomaterial conduits, and immunomodulation to sustain a proremyelination M2 environment—validated through remyelination‐specific biomarkers and powered by adaptive trial designs, are likely to yield the transformative improvements in functional recovery that single‐modality approaches have failed to achieve.

Understanding of peripheral nerve remyelination is at a turning point. The convergence of single‐cell transcriptomics, spatial genomics, advanced biomaterials, and gene therapy technologies creates unprecedented opportunities for translating mechanistic insights into transformative clinical therapies. The transcriptional hierarchy governing SC myelination—SOX10 pioneering chromatin accessibility, OCT6 bridging to KROX20, and KROX20/EGR2 commanding the compact myelin gene program—provides multiple nodes for therapeutic intervention. The NMNAT2–SARM1‐mediated axon degeneration cascade repositions Wallerian degeneration as an active, druggable self‐destruction program, while the discovery of SC senescence as a core mechanism of age‐related remyelination failure opens new avenues with clinically available senolytic drugs. It is anticipated that combination approaches simultaneously targeting the intrinsic SC myelination program, axonal NRG1 signaling, immunomodulation, and the physical environment of nerve repair will be required to achieve complete functional recovery after severe PNI.

## Author Contributions

Li‑Xin Huang, Tao Sun, Yi‑Bo Zhao, and Zhi‑Min Wu conducted literature collection, data integration, drafted the initial manuscript, and prepared all figures and tables. Jun Sun, Ming‑Yang Li, Qing‑Yi Huo, Yun‑Mou Ou, Qing‑Tian Liang, Cong Ling, and Bao‑Yu Zhang provided intellectual input, revised the manuscript critically, and verified key references. Hui Wang, Ying Guo, and Chuan Chen conceptualized and supervised the review, secured funding, revised the manuscript extensively, and approved the final version for submission. All authors have read and approved the final manuscript and agree to be accountable for all aspects of the work.

## Funding

Our research was funded by the Sichuan Science and Technology Program (Grant No. 2026NSFSC1628) Research Fund of Sichuan Academy of Medical Sciences and Sichuan Provincial People's Hospital (Grant No. 25QNPY044) National Natural Science Foundation of China (Grant Nos. 81571202, 82602674) Clinical High‐tech Projects in Guangzhou Area (Grant No. 20247‐GX30) Clinical Research 5010 Program of Sun Yat‐Sen University (Grant No. 2021001) 2024 Guangdong Huizhou Joint Fund Regional Cultivation Project (Grant No. 2024A1515140177) “Five‐Five” Project Construction Project of the Third Hospital of Sun Yat‐sen University (Grant No. 2023WW504) China Scholarship Council(Grant No. 202506380239) Clinical Research Program of The Third Affiliated Hospital of Sun Yat‐Sen University (Grant No. QHYH202505).

## Ethics Statement

The authors have nothing to report.

## Conflicts of Interest

The authors declare no conflicts of interest.

## Data Availability

The authors have nothing to report.
